# The experiences of three women who underwent termination of pregnancy for medical reasons: a comparative methodological study using grounded theory analysis and interpretative phenomenological analysis

**DOI:** 10.3389/fpsyt.2026.1840970

**Published:** 2026-07-08

**Authors:** Laura Sambrook, Rebecca E. Fellows, Elana Payne, Karen Burgess, Claire Storey, Munira Oza, The PUDDLES UK Collaboration, Flora E. Kent-Nye, Sergio A. Silverio

**Affiliations:** 1 School of Psychology, Faculty of Health, Innovation, Technology and Science, Liverpool John Moores University, Liverpool, United Kingdom; 2 Division of Methodologies, Florence Nightingale Faculty of Nursing, Midwifery & Palliative Care, King’s College London, London, United Kingdom; 3 Department of Women & Children’s Health, School of Life Course & Population Health Sciences, King’s College London, London, United Kingdom; 4 Department of Psychology, Institute of Population Health, University of Liverpool, Liverpool, United Kingdom; 5 PETALS: The Baby Loss Counselling Charity, Cambridge, United Kingdom; 6 Patient and Public Involvement and Engagement Group for Perinatal Bereavement, Trauma, & Loss, Department of Women & Children’s Health, King’s College London, London, United Kingdom; 7 The Ectopic Pregnancy Trust, London, United Kingdom

**Keywords:** abortion, grounded theory analysis, interpretative phenomenological analysis, mothers, perinatal bereavement, qualitative research, termination of pregnancy, women

## Abstract

**Introduction:**

Termination of pregnancy [ToP] for medical reasons, especially for a wanted pregnancy, is an underexplored area in public health. Women face significant emotional, psychological, and social challenges, often compounded by inconsistent policies, healthcare inequities, and societal stigma. Additionally, the decision-making process can be influenced by financial and systemic factors, with women reporting long-lasting emotional impacts and inadequate mental health support following a ToP.

**Methods:**

Semi-structured interviews were conducted with three women who underwent ToP for medical reasons, for an otherwise wanted pregnancy. Data were analysed using Grounded Theory Analysis [GTA] and Interpretative Phenomenological Analysis [IPA] to explore the personal experiences of women who underwent ToP, focusing on their grief, decision-making, and interactions with healthcare professionals.

**Results:**

Three key themes emerged from the GTA: (1) terminating a wanted pregnancy; (2) compassionate terminology; and (3) lasting impact. Together these led to the emergent theory of ‘A Shadow Not Shaken’. The IPA also rendered three key themes: (1) holding onto anger; (2) terminology undermines pain; and (3) advocating for self. The GTA focused mostly on the psycho-social experiences of their interactions with healthcare professionals and how this shaped their perception of their ToP, whilst the IPA provided an understanding of how these interactions were experienced.

**Discussion:**

This study highlights the emotional impact and lasting effects of ToP for medical reasons. It emphasises the need for compassionate, empathetic care from healthcare professionals to reduce stigma and psychological distress. Healthcare providers should prioritise emotional support and clear communication. Future research should explore how these results apply to diverse contexts, aiming to improve care and reduce stigma for women undergoing ToP. Clinical practice should reflect how the relationship between women and healthcare professionals is crucial, and account for the current lack of empathetic care, adequate emotional support, and ongoing stigma relating to ToP. The study also provides further evidence for the value of using dual-methodology qualitative approaches.

## 1 Introduction

Termination of pregnancy [ToP] continues to be a neglected area of public health research, with women’s mental health and psychological well-being often disregarded when it comes to the decision-making process, the procedure itself, and the aftermath of both these factors. There is a lack of unified policy across the globe with respect to the management of ToP (1, 2) – especially true throughout Europe and North America where health inequalities and general inequity in access to healthcare are widening – exacerbated by changing political landscapes (3), including increasingly polarised Governments (4, 5), changes to European Union member states (6), and of course, the global pandemic (7).

In 2021, of the 214,256 terminations of pregnancy reported within England and Wales, 1.6% were performed due to the significant risk posed that a child born may suffer from life-disabling conditions (8). These risks are often discovered during routine antenatal screening, as is standard practice within maternity care, following which individuals may be faced with an oftentimes extremely difficult decision to make about the continuation of a pregnancy (9). The decision-making approach of individuals has been found to be greatly influenced by the existing clinician-patient relationship, whereby patients may feel anywhere between empowered and supported, to disregarded and isolated based on their healthcare interactions (9), and their ability to generate candidacy in healthcare settings (10). Aspects of candidacy and related advocacy and empowerment studies have been especially useful in unpacking the decision-making processes by women regarding a ToP (11, 12); their access to and utilisation of abortion care services (13, 14); and their subsequent perspectives of their decision and the ToP procedure itself (12, 15).

Furthermore, it is evident that weighing up such a decision can go beyond the direct matter of the condition itself. Women who report having a stable life situation often report feeling their decision to undergo a ToP procedure for medical reasons was influenced by a lack of financial and systemic support they perceived to be required and not in response to societal judgement (16). Additionally, a recent review has demonstrated women who undergo a ToP for medical reasons experience a plethora of negative emotional responses which could be negated by having the support of close friends and family; and engagement with shared decision-making, leading to less severe, long-term psychological outcomes (17). A recent report found that 38% of women who underwent a medical ToP engaged in mental health services, with 55% wishing they had received support during a subsequent pregnancy (18). This provides clear evidence for the fact that women who have sought a termination due to medical reasons, may well experience long-lasting impacts directly relating to their care experiences, and which may have an effect on reproductive health planning, subsequent pregnancies, and/or future ToPs they may choose or may have to have.

It is important to note that at times, medical professionals may encounter patients who decline such terminations. Reasons for this may relate to feelings of guilt, religious beliefs and ideologies, or a mistrust in the diagnosis or prognosis shared with them (19). In these situations, healthcare professionals may be presented with a scenario whereby they may have to respect a patients’ decision despite the fact that this may not be congruent with their personal knowledge and beliefs, or the patient’s well-being (20, 21). Nevertheless, this emphasises the importance of delivering patient-centred care whereby women are well-informed and listened to in all aspects of their care (22). Further studies have highlighted these tensions in ToP-related care (23) including how personal experience of ToP has been found to change women’s opinions about termination, as well as modifying any further reproductive plans (24). From this it is of clear importance that women develop and/or maintain an empowered view of ToP and are supported in their decision-making so they can continue to make the correct reproductive health decisions for them and in-line with their health, psychological, social, and moral wishes. Further, it is important to empower women with informed decision-making and the impact of care when undergoing a ToP, on future pregnancies and associated care. Given the reasons above, further research exploring the psychological effects and psycho-social experiences of undergoing a ToP procedure is imperative.

## 2 Methods

### 2.1 The present study

This analysis presented herein is derived from a wider, international, qualitative study called PUDDLES (25–30). In the present analysis, we opted to undertake a dedicated exploration of the experiences of three women who had undergone a ToP of an otherwise wanted pregnancy; by utilising two qualitative methodologies. We therefore ensured we were accurately interpreting data from participants who were under-represented in the wider PUDDLES study dataset: women who had undergone a ToP. We were determined to do so, as this group of women’s voices are so seldom heard, and this way of analysing and interpreting the data provides a platform from which these women could narrate their own lifecourses (22).

### 2.2 Ethical approvals

Ethics approval for PUDDLES-UK studies were sought and granted from the King’s College London Research Ethics Committee [refs:- HR-19/20-19455, June 2020; HR/DP-21/22-28808, March 2022].

### 2.3 Patient and public involvement and engagement

The PUDDLES Study has been conducted in collaboration with a number of charities with which it has partnered (PETALS: The Baby Loss Counselling Charity; The Ectopic Pregnancy Trust) and from which it has received input (The International Stillbirth Alliance; Tommy’s Charity; Sands; British Pregnancy Advisory Service). Furthermore, The PUDDLES Study has sought advice on every aspect of the study – from design to dissemination – from patients; experts by experience and those with lived experience; lay members and members of the public; academics, researchers, clinicians, and policy makers; as well as parent advocates, charities, and other third sector organisations across a number of meetings. These have been hosted and facilitated by the National Institute of Health and Care Research Applied Research Collaboration – South London [NIHR ARC-SL] Maternity and Perinatal Mental Health Research Theme PPIE Group and an NIHR ARC-SL Work in Progress Meeting; the King’s College London Department of Women & Children’s Health PPIE Group for Perinatal Bereavement, Trauma, & Loss; the Chief Midwifery Officer and the Maternity Transformation Team of NHS England and NHS Improvement; and through the PIVOT-AL National Collaborative for Maternal and Child Health Research during the Pandemic.

### 2.4 Theoretical approach

Adopting a lifecourse approach allowed us to situate the phenomenon of interest (the experience of a ToP) as a lifecourse rupture – a lifecourse transition which changes the trajectory of one’s life, and in turn can be seen as an empirical site of inquiry (31). We therefore underpinned our analysis philosophically with a critical realist ontology and an objectivist epistemology, rendering the study paradigmatically post-positive. In practice, this meant we were acceptant of participant’s narratives as a form of ‘lived reality’, even if the narratives provided in the interviews themselves were only representative interpretations of the events on which they were reporting and not the whole, factual truth. In terms of positionality, our reflexive judgement was empathic (or critical) – accepting of behaviours and perceptions being malleable in response to societal norms and expectations. We also adopted a mixed subjective-spectator/objective-outsider position within the data – given that some of the authorship team and the wider PUDDLES team have clinical experience of ToP procedures, whilst others have not.

### 2.5 Participants and data collection

This paper represents an experimental qualitative study pitting two methodologies against one another to see how they cope with a purposefully small sample size. In this study we present analyses based on three women who had undergone a ToP. Given the ‘N of 3’ nature of this study and the potential for identifiability, we do not present any further demographic information relating to these women, other than they underwent their ToP procedure.

Interviews were in-depth and semi-structured in nature conducted using video-conferencing November and December 2020 (The PUDDLES Study; n=1) and between March and June 2022 (The PUDDLES – Early Pregnancy Loss Study; n=2). The interviews were conducted by an experienced qualitative researcher (SAS) with expertise in undertaking interviews on sensitive topics; and a Master’s student (FEK-N) who had been trained to an advanced level of qualitative research and analysis. Data collection adhered to protocolised best practices for field research of a qualitative nature (32). The interview schedules were informed by a cross-disciplinary team of academics, clinicians, and members of charitable organizations in the field, and have previously been published elsewhere (26, 27). The semi-structured nature of the interviews allowed for commonality of questioning across all participants, but enough flex in the interview schedule to allow for spontaneous conversation and follow-up of points pertinent to individual participants (33).

### 2.6 Data analysis

Given the experimental nature of this empirical endeavour, we interrogated data using both Grounded Theory Analysis [GTA] and Interpretative Phenomenological Analysis [IPA]. We prioritised the GTA to safeguard the requirement for no *a priori* assumptions necessary for GTA methodology. IPA is often indicated as the (only) analytic methodology which can cope with a *small-n* sample size, however, recent years have seen efforts made to highlight how GTA can have similar, if not better utility in the case of *small-n* research settings. We therefore offer a comparative dual-analysis allowing for interpretation to be made as a result of each methodology, and aid in the clarification of findings based on analytic rigour associated with triangulation between methodologies, whilst auditing the process to enhance analytic transparency. Analyses were undertaken and completed by one researcher (LS) who had not been part of the data collection team. Data analysis was appropriately iterative and inductive, with the element of rigour maintained through regular, consultative meetings with the study’s Chief Investigator (SAS). Upon completion of analyses, results were subject to comparative interpretation, which exemplified similarities and differences between the findings from the two analytic methodologies.

#### 2.6.1 Grounded theory analysis

The three transcripts were first analysed using Grounded Theory (34), ensuring we adhered to the need for a *‘tabula rasa’* when using this methodology. Transcripts were iteratively coded by hand following a participant-by-participant process, whereby the first transcript was analysed in full before the second transcript was analysed, and this was completed before the third and final transcript was analysed. In terms of coding, transcripts were first coded using verbatim (‘line-by-line’ or ‘sentence-by-sentence’) codes; then using descriptive (‘focused’) codes; then analytic codes (‘super-categories’) which were a result of merging descriptive codes together; and finally interpretive codes (‘themes’) were generated through the process of collapsing, splitting, or re-organising super-categories. As with all Grounded Theory Analyses, the theory emerged from detailing the relationship between the themes (35). Comparisons were drawn within and between transcripts which was important for the maintenance of analytic rigour and accuracy in analytic interpretation. Analysis led to the development of three themes, each a result of the clustering of a number of super-categories.

#### 2.6.2 Interpretative phenomenological analysis

All three transcripts were subsequently analysed using Interpretative Phenomenological Analysis (36), which is a systemic qualitative analysis whereby participant narratives can be extracted into themes and interpreted. In this analysis, there were three superordinate categories created from the recurring themes present in the interviews. These superordinate categories comprised smaller, more precise, emergent, subordinate themes. The transcripts were analysed in turn, with initial analytic thoughts noted in the margin of each transcript, where the emergent themes were also documented. These themes were often representative of key words which captured the essence of the data. Codes were then listed, grouped into subordinate themes, and then then finally organised into superordinate themes. Once this was completed for the first interview, the process was repeated for the second, and then the third, in a process of thematic discovery across all transcripts. The process relies on being cyclical (37), with all new themes being tested against previous ones, and new transcripts being compared to those which had already been coded.

## 3 Results

### 3.1 Grounded theory analysis

**Figure 1:**
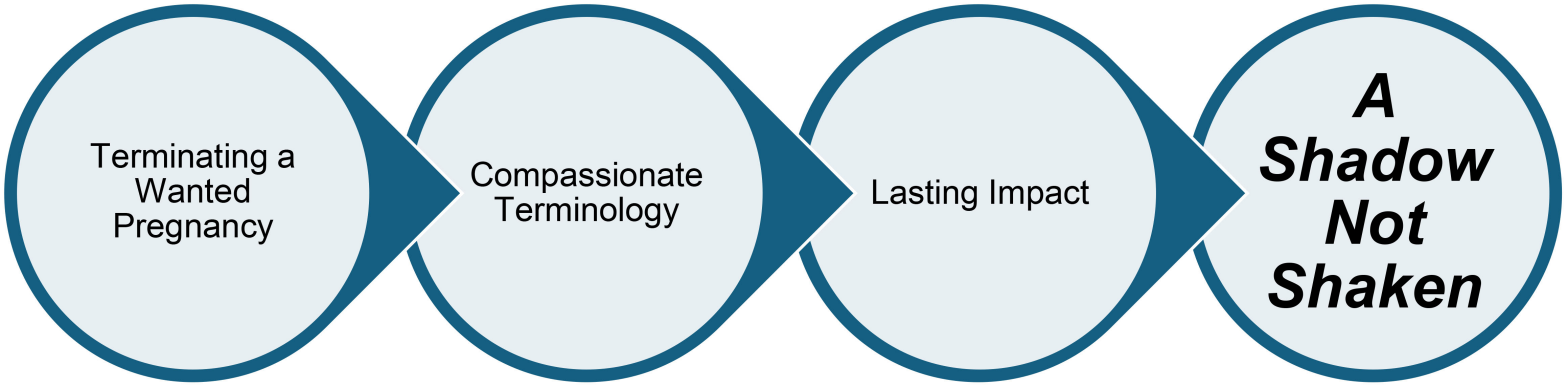
Thematic diagram of GTA themes and theory.

Using data from all three participants, three themes emerged from the collapsing, merging, and/or splitting of ten super-categories (see **Table 1**): ‘Terminating a Wanted Pregnancy’ (lack of understanding, stigma around termination, making the choice for them); ‘Compassionate Terminology’ (resentment towards medical language, foetus vs. our baby, terminology as a trigger); and ‘Lasting Impact’ (unimaginable pain, ruptured life plan, self-blame, impact of loss on relationships), see **Figure 1**. The findings of each theme are presented first, followed by an interpretation of theory which describes inter-relationships between themes (35). Illustrative quotations from all participants are included in the narrative to evidence key components of the theory.

**Table 1:** A comparative table of lower-order and higher-order themes from the GTA and IPA analyses.

Grounded theory analysis		Interpretative phenomenological analysis	
Super-categories (lower-order themes)	Themes (higher-order themes)	Superordinate themes (higher-order themes)	Subordinate themes (lower-order themes)
Lack of understanding Stigma around termination Making the choice for them	Terminating a Wanted Pregnancy	Holding onto Anger	NHS failingsNegative staff attitudes Lack of expectation setting
Terminology as a trigger Foetus vs. our baby Resentment towards medical language	Compassionate Terminology	Terminology Undermines Pain	Overly medical language Stigma around termination Terminology as a trigger Baby deserves acknowledgement The pain of leaving hospital empty-handed Unrealistic expectations of grief
Unimaginable pain Ruptured life plan Self-blame Impact of loss on relationships	Lasting Impact	Advocating for Self	Fighting for self Having to find own answers Onus on mother A no-choice choice Questioning belief system Protecting self from judgement

#### 3.1.1 Theme 1: terminating a wanted pregnancy

In addition to managing their own grief, women had to manage the expectations and opinions of loved ones. They encountered unhelpful attitudes and misconceptions around their experiences (lack of understanding) and judgement (stigma around termination), but ultimately felt they had made their decision for the right reasons, as they had chosen to protect their child from harm (making the choice for them).

Women were open and honest with their loved ones about the loss of their baby, but were disappointed that their responses lacked understanding about the reality of the grieving process. There appeared to be a social expectation with which women were confronted whereby in both personal and professional contexts, the lack of support, understanding, and general awareness around ToP was coupled with unhelpful expectations and pressure to ‘move on’ quickly. In some instances, managers expected the women to continue to work as normal after their loss, whilst friends expected any grief to be short-lived:

“I think with friends, that because they’ve not gone through it themselves, they don’t really understand, so it’s like they expect you to be sad for a couple of weeks and then be okay. And it’s not like that.” – Penelope.

Women were understanding and highlighted it is difficult to grasp the extent of the pain resulting from baby loss without having lived experience of it, and were happy to educate loved ones on the meaning of termination for medical reasons. Women’s motivations for educating others were often centred on reducing stigma, catharsis, and moving through grief by discussing their experiences openly:

“I just want it to be understood that not all babies come home. I think that’s my thing about baby loss.” – Molly.

Women associated the stigma they experienced with a lack of understanding. They felt that awareness needed to be raised around baby loss and termination for medical reasons. They reported experiencing fear about being judged by loved ones, with one mother explaining that her siblings felt she should not have terminated her pregnancy, as it was ‘just Down’s.’ Women found it difficult to discuss the details around their termination, especially where they perceived stigma around medical termination compared to other forms of pregnancy loss:

“I think with the termination, I think because there’s a stigma about it, I just kind of lump that in, unless it’s people that know me very well, it’s something that I lump in with the others as a loss.” – Penelope.

However, women shared the opinion that they wanted to celebrate the short lives of their babies and felt they should not be denied this due to a lack of understanding and stigma around the topic:

“People need to know about it. It needs to be less of a taboo subject, because why should I feel on edge talking about my baby, to save the feelings of others?” – Molly.

Women wanted it to be known that, although they had made the decision to terminate their pregnancy, they had done this with their child’s best interests and/or wellbeing at heart. Medical professionals had identified that their baby had serious health complications that would impact them for the rest of their lives, or perhaps prevent them from surviving at all. Women highlighted that their babies had been ‘very much wanted’ but that, ultimately, they made the decision to terminate to protect them from pain and suffering:

“We knew that, if we could avoid it, we didn’t want to bring a child into the world who was potentially going to have a lifetime of problems as a result of us wanting to have a baby. We made the choice for him rather than ourselves, so we know we made the right decision.” – Tia.

Women explained that concerns around their baby’s health and wellbeing were central to their decision making. They were not only worried about their baby’s physical health, but also considered the potential social difficulties and negative impacts on wellbeing that their child may experience as a consequence of their condition:

“We didn’t want to put her at risk of multiple surgeries, exclusion, the lack of understanding of diversity in the world that we live in, and potentially what else could lie ahead of her.” – Molly.

#### 3.1.2 Theme 2: compassionate terminology

All participants discussed the negative impact language can have on a woman who has terminated a pregnancy for medical reasons, highlighting the need for staff to use compassionate terminology. Women felt doctors lacked empathy for this form of early pregnancy loss, using overly medical language when referring to their baby (resentment towards medical language) and refusing to acknowledge their baby as a human being (foetus vs. our baby), which could feel triggering (terminology as a trigger).

Women felt the choice of language used by doctors was unnecessarily medical and did not take into account their grief. They reported resentment about their loss being referred to as a termination when they preferred to view it as a stillbirth:

“I mentally class it as a stillbirth because I resent the fact that doctors refer to it as a termination, because we terminated because of a medical issue that happened. It wasn’t a choice to terminate in the respects of any other choice.” – Tia.

Women reported that the use of medicalised language resulted in doctors appearing cold and lacking in empathy. In medical terms, they understood that what they had experienced was technically a terminated pregnancy; however, they felt this terminology simplified their experience and did not consider the difficult decisions they had had to make to reach that point:

“‘Well, what treatment did you have?’ Again, it comes back to that terminology. It’s not a treatment. It wasn’t a treatment, far from it. I wasn’t at a day spa. I delivered my baby that I was never going to bring home.” – Molly.

Women reported finding it distressing when hospital staff referred to their baby as a ‘foetus,’ or worse, ‘retained products of conception.’ They wanted it to be recognised that they had loved their baby (and held it when the opportunity presented itself), and that their child deserved to be acknowledged as a human being, rather than being viewed through a medicalised lens or simply as a medical event:

“I think they look at baby loss, especially if it’s only early days of a pregnancy, it doesn’t really matter. Again, it’s a foetus, a bundle of cells, that’s what you see it referred to. Actually, it’s not, it was my baby and [baby name] really was a baby. We held her, we saw her, we felt her.” – Molly.

Participants highlighted both the physical and emotional impact of this form of loss, wanting this experience to be acknowledged and for a clearer distinction to be made between termination of pregnancy and other forms of pregnancy loss:

“The fact that I had to go into hospital and deliver my baby, I couldn’t have done it at home – and I’ve had natural miscarriages – to refer to that as a miscarriage is insulting because you are being made to deliver your baby. That is a birth.” – Tia.

Women felt the use of medical and cold language was not only distressing but triggering. They felt that referring to their loss as a termination suggested they wanted to end their baby’s life and did not account for the struggle they had experienced when making this decision:

“Termination cuts quite raw, when you feel like you’ve had no choice, to be told that you made a choice is a bit brutal.” – Tia.

Women recounted the trauma of choosing to terminate their pregnancies for medical reasons, in the hope of preventing their child from a life of suffering. They wanted this to be recognised through the terminology used in their care:

“It wasn’t a choice for us. It’s a no-choice choice. We didn’t want her suffering, hurting.” – Molly.

#### 3.1.3 Theme 3: lasting impact

Women described their lives following the loss of their baby. They discussed their intense grief and heartbreak, which was heightened by times of isolation (unimaginable pain) and discussed how they had struggled to come to terms with their change in circumstances (ruptured life plan). They reported experiencing not only grief but guilt (self-blame) and felt their loss had changed the nature of their relationships with loved ones (impact on relationships with loved ones).

Women reported that the pain they experienced following the loss of their baby was unlike anything they had felt before. Even women who had experienced previous miscarriages explained that the pain of terminating a wanted pregnancy was different:

“We unfortunately had been used to heartbreak and disappointment. We hadn’t quite appreciated that it could happen so late, so that was a whole new level of pain.” – Tia.

They explained how seeing other women with their babies who had survived intensified their pain, recommending the need for a specialised maternity area for those who have lost their baby:

“You’re watching happy families when your whole world has imploded.” – Molly.

Women explained how difficult they found it to come to terms with the rupturing of their life plan. They had imagined a life with their baby and an image of who they would be as a mother, only to have to accept that this was no longer the reality:

“I think for me and other baby loss parents, you just want to scream. I was only 17 weeks, yes, fine, but it was still my baby. I’d still set out a life plan for us.” – Molly.

They felt their loss would impact their future experience of pregnancy, if they ever felt strong enough to try again:

“I think that, if we did move forward and have children or planned to try and have children, I can’t find myself having any enjoyment in it. I think I’d be a very anxious wreck, even going for that first scan.” – Molly.

Women reported finding it difficult to forgive themselves for the loss of their baby, despite having been reassured by medical professionals that it was not their fault. They experienced ongoing guilt and self-blame in a way their non-gestational partner did not:

“It was my body that, in my mind, failed.” – Molly.

Women found themselves questioning their choices throughout the pregnancy, worrying that their actions might have contributed to the outcome:

“That’s the thing that’s still in the back of my mind, that guilt that we could have prevented it. But that’s probably the biggest emotion I still carry.” – Tia.

Understandably, women reported that the loss of their baby had impacted their relationships with loved ones; however, some reported closer familial relationships whilst some reported distancing themselves from others:

“Things have definitely been difficult with me and my partner. He’s been incredible and supportive and stuff. But when you do have a loss and your hormones are all over the place and you’re not exactly full of the joys of spring and you’re crying and maybe self-medicating with a bit of wine here and there, you’re not necessarily the easiest person to live with.” – Penelope.

“It’s probably brought my parents and I closer together. We used to see each other every now and again and now we sort of talk every couple of days and we Zoom and we do all sorts of things.” – Penelope.

### 3.2 Interpretative phenomenological analysis

**Figure 2:**
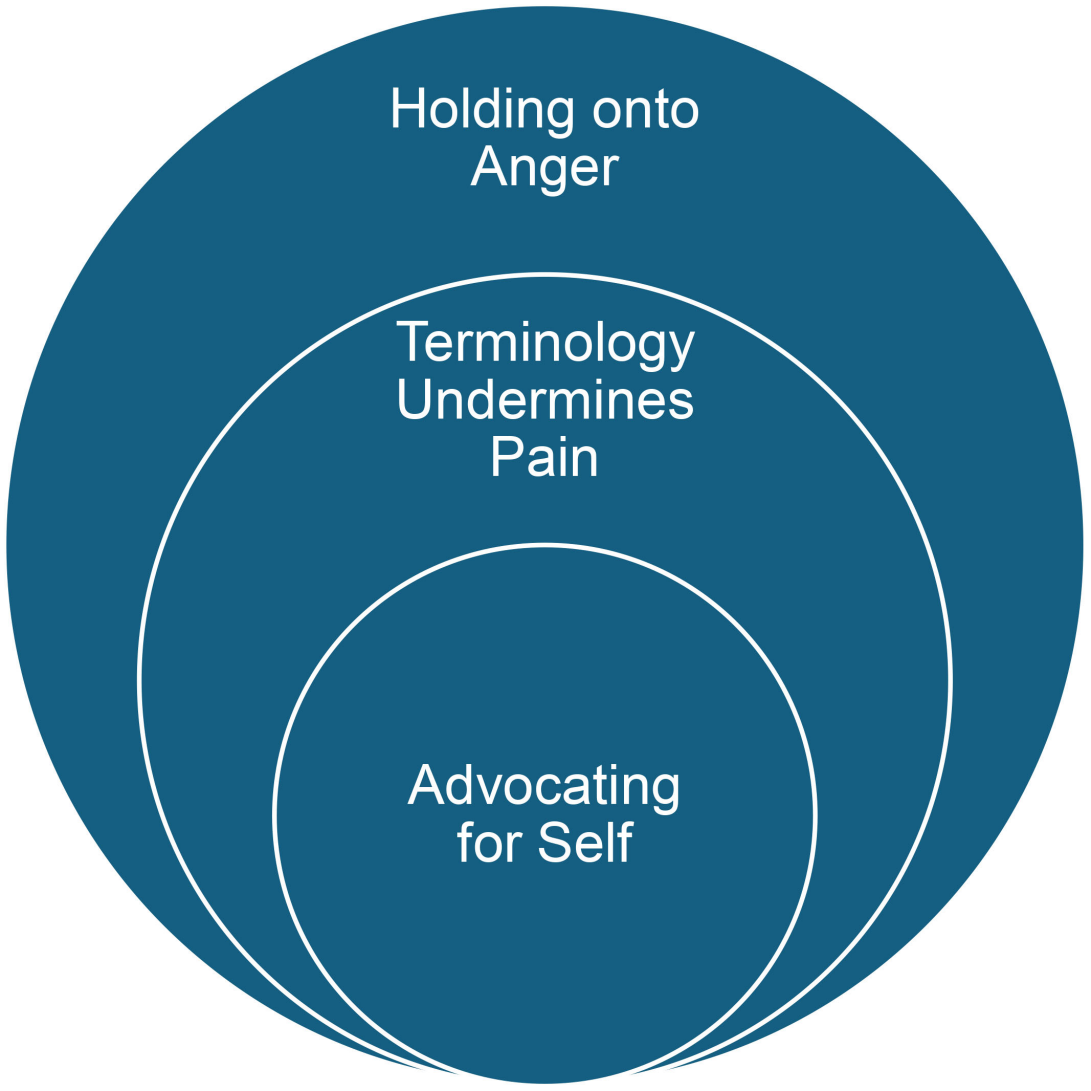
Thematic diagram of IPA themes.

The analysis yielded 18 emergent themes in total (see **Table 1**), which were condensed into three superordinate themes which conceptualised the experiences of women who had experienced a termination for medical reasons: Holding onto Anger (NHS failings; Negative staff attitudes; Lack of expectation setting); Terminology Undermines Pain (Overly medical language; Stigma around termination; Terminology as a trigger; Baby deserves acknowledgement; The pain of leaving hospital empty-handed; Unrealistic expectations of grief); and Advocating for Self (Fighting for self; Having to find own answers; Onus on mother; A no-choice choice; Questioning belief system; Protecting self from judgement). Each superordinate theme had several corresponding sub-ordinate themes (see **Figure 2**).

#### 3.2.1 Superordinate theme 1: holding onto anger

When describing their experiences of maternity care for baby loss, women reported an undercurrent of anger towards the NHS and its staff. They highlighted failings which they felt had contributed to their trauma, alongside areas for improvement, evidencing a need to improve care for future bereaved parents. Women described a pattern of losing faith in the NHS, ranging from frustration about the unwillingness of consultants to conduct further tests after a loss, to outrage at medical negligence:

“It turned out I had retained products. Come on, even without a medical degree, as the doctor shoved in my face, retained products for almost two months since I’d had the baby, it doesn’t sound very good, does it?” – Molly.

Although there were instances when women were provided with outstanding maternity care, there were also times when staff responsible for their care presented as cold, unempathetic and disinterested. Women reported feeling that they had to fight for the treatment they felt they deserved, with staff failing to measure up to the standard of expected care:

“This is the only thing I get really upset about … [crying] It was the foot and handprints. I was told, ‘No, you won’t get them at this stage,’ which I know is a lie because I know people who have had them done and I didn’t get them. [ … ] And it’s the one bit I am a bit hostile about. But I trusted them, they were the ones in the know. They knew, they didn’t even try.” – Molly.

Women reported feeling angry that they had placed their trust in NHS staff and felt that this had been misplaced. It was during these discussions about misinformation and broken trust, women became most upset, highlighting how these emotions were difficult to let go. Women felt they should have had their expectations set by the staff responsible for their care, highlighting a lack of information giving during a time where they felt scared and did not know how to prepare for their birth and its after-effects:

“I was not given much information, to be honest. I was a first-time mum, didn’t know what to expect. It wasn’t discussed what would happen physically, that my waters would break. I wasn’t told about the placenta.” – Molly.

“I wish they would have told me because yes, it still could have happened, but I might have been able to prevent it. So that was incredibly frustrating, that I had gone through so much and they clearly could see that I was an intelligent, researched human being, capable of understanding the risks of a pregnancy and no one bothered telling me.” – Tia.

Women felt that not only was there an overall lack of compassionate care, but that aftercare was non-existent. They reported that they would have appreciated ongoing emotional support or even a follow-up call after the loss of their baby, but that this did not happen:

“Emotionally, we got nothing as well. So, I would probably say some follow-up care would be nice [laughs] – anything. We got absolutely nothing.” – Tia.

#### 3.2.2 Superordinate theme 2: terminology undermines pain

Women called for a change to the language used to describe baby loss when undergoing maternity care. They felt the terminology used was overly medical and dismissive, and that it perpetuated the pre-existing stigma around termination:

“The only people I found cold were the doctors when we delivered, who do the rounds. So, obviously you’re mostly with midwife care, but the doctors doing their rounds were quite old and kept referring to it as a termination. Yes, by all intents and purposes, medically that was, but it wasn’t a termination because of any other reason than we felt we had to.” – Tia.

Women highlighted that there is a lack of understanding about termination for medical reasons and that they had been fearful of sharing their news with loved ones for fear of being judged; however, they had decided to speak openly about their loss in order to celebrate the life of their baby. Women felt their baby deserved the same level of acknowledgement as a child who had lived, and found the medicalised language and terminology used by doctors triggering, as they felt it undermined the pain they had experienced:

“The fact that I had to go into hospital and deliver my baby, I couldn’t have done it at home – and I’ve had natural miscarriages – to refer to that as a miscarriage is insulting because you are being made to deliver your baby. That is a birth.” – Tia.

Women were met with a lack of understanding and compassion regarding their loss. Women felt as though ‘people have needed educating’ on what a termination for medical reasons is, and that their baby was more than ‘a bundle of cells’:

“For me, I wanted to be honest and open because I want to celebrate her life. She’s part of me, part of my history and my life, and I don’t want to shy away from her and hide her away, just because of us choosing to end her life.” – Molly.

It was important for women to raise awareness of the pain they had experienced following their loss, as some of their loved ones had struggled to understand the extent of the grieving process:

“They don’t really understand, so it’s like they expect you to be sad for a couple of weeks and then be okay. And it’s not like that. You don’t lose your husband and then be okay in a couple of weeks’ time, so I think that it’s not really understood.” – Penelope.

Women provided harrowing descriptions of leaving the hospital without their baby, with one describing how she left ‘clutching a little teddy bear’ instead of her child:

“Twice I’ve left that building feeling very sad and low, the first being when I walked out with just a box, a memory box, and there’s a mum and dad taking their baby home for the first time, and I was taking a memory box through the same doors. I’m not expecting massive airs and graces, but just to be aware that exiting that building without your baby hurts a hell of a lot.” – Molly.

Although their grief was unavoidable, women noted ‘little touches’ and keepsakes provided by midwives provided comfort during a time of unimaginable pain:

“They were so kind. They gave us a little memory box with footprints and handprints and a little certificate. It’s not a birth certificate but like an acknowledgement that your baby was there.” – Penelope.

#### 3.2.3 Superordinate theme 3: advocating for self

Women reported feeling abandoned by maternity services in relation to information giving, follow-up care and the possibility of future testing. They felt the onus was on them to do their own research into baby loss and subsequent care, as nobody was advocating for them:

“Every day I checked my temperature because that’s a sign of an internal infection and after birth you should be really careful, but this is all based on my own readings. This is based on nothing a doctor or midwife I physically saw gave me practical advice on. And emotionally we got nothing as well.” – Tia.

They highlighted the frustration they felt when trying to advocate for themselves in relation to the care they believed they deserved. They felt they were dismissed as they had chosen to terminate their pregnancy, despite it feeling like a ‘no-choice choice’ due to their baby’s poor prognosis:

“I was like, ‘I would like to have every single test you can possibly give me, make sure you give me this test’ and I had to fight to have tests, because in their head, I’d terminated. It wasn’t anything that was wrong with me or the baby, I’d chosen to terminate.” – Tia.

“It’s a no-choice choice. We didn’t want her suffering, hurting.” – Molly.

Women reported using their voices and fighting for themselves as a way to protect themselves from the judgement of others. Although they knew they had made the right choice for the health and wellbeing of their child, they were aware of the stigma associated with terminating a pregnancy and, at times, even questioned their own decision:

“The post-mortem did confirm the diagnosis, so when it came, it did give massive comfort, because at least we knew that we’d made the right decision. Yes. It was kind of good because you sort of start questioning yourself. You’re like, did I? Should I do it? Would it have been okay?” – Penelope.

## 4 Discussion

### 4.1 Summary of main findings & interpretation

Using two different qualitative methodologies to analyse the accounts of the women recruited to The PUDDLES Study provides a unique insight and opportunity to truly hear the voices of women who suffered the grief of a termination of pregnancy for medical reasons and gain an understanding of their painful lifecourse transitions from expectant to bereaved parents. There were bound to be considerable similarities in the findings of both analyses due to only having access to three accounts (although this is considered a reasonable sample size for IPA); however, **Table 1** evidences considerable differences in the content of the themes created.

In terms of commonalities, both the GTA and IPA findings highlighted the impact of negative language used by care providers on the women’s emotional wellbeing and advocated for the use of more compassionate terminology. Women wanted to be recognised as mothers who had lost a child, however, we recognise will not be for other women who seek ToP procedures. The GTA focused mostly on the experience of being spoken to without compassion, whilst the IPA was able to elucidate the anger and frustration the women felt about their grief being undermined and the stigma of termination perpetuated by staff. The impact of cold and medicalised language and lack of empathetic and compassionate care by staff often led to conflict between the woman and her medical team leaving her with negative experiences of care.

Termination of pregnancy for medical reasons is undoubtedly an emotionally painful situation (38), which requires compassionate care, free of judgement (39). Women have previously reported clear recollections of interactions with care providers – words which either caused further distress or provided great solace – and which their distress was exacerbated when information was provided without overt sympathy and acknowledgement of the enormity of the decision to undergo a ToP (40). It is evident from both our research and other studies that some women require acknowledgement that a pregnancy terminated for medical reasons is a baby they wish to be recognised for having existed, and in these cases their loss was tangible and real, and they require respectful and dignified interactions, perceived as vital by other bereaved mothers (41).

Another commonality noted in both the GTA and IPA findings was the initial conflict women experienced when deciding whether or not to terminate their pregnancy. As demonstrated in the literature, this decision is both powerful and paradoxical in nature, and can result in deep emotional pain and conflict for women (41). This sentiment has been argued by others who have suggested women can struggle with presiding over both the potential to give life and the decision to end it (42). When faced with an ethical dilemma with two poor alternatives: either to continue a wanted pregnancy and potentially cause suffering to the child or terminate the pregnancy and experience subsequent guilt, the women in this study chose to spare their child any unnecessary pain. Support from their partner, loved ones and care providers has been associated with greater confidence in decision-making and a positive psychological impact (43).

Both findings acknowledged the stigma associated with ToP; however, the GTA was more concerned with managing the unhelpful attitudes and opinions of others alongside the women’s grief, whilst the IPA highlighted the need for women to protect themselves from judgement by fiercely advocating for themselves and future bereaved parents. Women described the social withdrawal associated with their loss, an identified expression of grief following perinatal bereavement (44, 45). Women felt they could not easily share their decision with others for fear of judgement, resulting in loneliness and vulnerability (46). In line with previous research, women appreciated having their decision validated by loved ones and health professionals (47).

The GTA allowed for the emergent theory: ‘A Shadow Not Shaken’, something we feel encapsulates not only the intense grief experienced after the termination, but the ruptured lifecourse, which can on occasion thwart the expectations, leading to loss of hope for the future – something we have seen in previous studies (45, 47). The IPA on the other hand – whilst not rendering a theory – painted a slightly alternative picture, with more focus on the technicalities of coping after a ToP, such as exploring NHS failures and advocating for future parents, rather than the experience of the rupture as a whole (as seen in the GTA). Within the IPA, women were concerned about the lack of expectation setting and aftercare provided by staff and felt this caused unnecessary stress. A lack of follow-up care and insufficient information about what to expect during birth has been noted in previous literature, with care often described with dissatisfaction in relation to factors such as insufficient pain relief (48, 49).

Both analyses highlighted the difficult emotions experienced by women in the aftermath of a ToP, with the GTA focusing on the support networks they relied upon and the practical methods they used to heal; whilst the IPA developed these concepts further, adding nuance to what support helped and how. This is something of which to be especially aware of when providing care for mothers between lost and subsequent pregnancies. The analyses enabled detailed exploration into the ways in which women made sense of their personal and social world and highlighted not only the way that they felt marginalised during their experience, but the impact this had on them as an individual within society. Whilst perinatal mental health has achieved deserved attention in recent years (22, 50), it is essential the supportive needs of these women are recognised, to provide an appropriate context for systematic and comprehensive interventions to support women during and after their termination. Care providers should come from a non-judgemental standpoint, with various studies recommending that health professionals should be better trained in the emotional impact of termination of pregnancy for medical reasons to enable them to provide quality care and advice. We should continue to learn from global research, policy, and practice, with the potential for reverse engineering of practices learnt from low- and middle-income countries to ensure ToP access remains accessible and decentralised (i.e. not impermeable to those who require or desire a ToP (51),) whilst ensuring access to reproductive health services does not rely on socio-economic status, but rather necessity and need (52). Further research which acknowledges and prioritises the female voice and the lived experience of women is required (41).

### 4.2 Strengths, limitations, and future directions

This study demonstrates methodological rigour by employing both Grounded Theory Analysis [GTA] and Interpretative Phenomenological Analysis [IPA] to examine the experiences of women who have terminated a pregnancy for medical reasons. The use of dual qualitative methodologies allowed for a deeper exploration of the women’s grief, the lasting psychological impact, and their relationships with healthcare providers. This methodological approach provides a more complex understanding of an under-researched area, particularly highlighting the need for compassionate care and improved communication in maternity services.

Although the sample size was small, it is important to acknowledge that *small-n* studies are widely accepted in IPA research, and this study adds value by amplifying the voices of women who have often been marginalised in perinatal bereavement research. This study contributes to a growing body of work that emphasises the importance of recognising women’s lived experiences, particularly in the emotionally charged context of medical termination, despite not adhering to traditional recruitment standards. We recognise the *small-n* nature of the study may limit generalizability and the lack of demographic detail which preserved anonymity, may reduce the contextual richness available to readers.

Clinical implications for policy and practice emanating from this study would be to ensure adequate training of healthcare professionals on the emotional impact of the termination to ensure it is adequately buffered by providing detailed information about the potential quality of life for the child to enable parents to make informed choices, whilst also empathising and being present with the mother, and validating the decision to terminate the pregnancy. Future research should seek to test the theory of ‘A Shadow Not Shaken’ identified in this study across different contexts, such as with larger and more diverse populations, in other types of perinatal loss such as early elective abortions, and in healthcare settings which do not operate on a ‘free-at-point-of-access’ system; to determine whether the findings hold under different circumstances; and work with larger datasets to see whether this nuanced understanding applies across women who undergo a ToP. Moreover, the insights into compassionate care and the stigma surrounding medical termination call for further investigation into how healthcare professionals can better support women who undergo this experience. A detailed methodological reflection on the comparison between GTA and IPA in *small-n* qualitative research is also needed to explore the strengths and utility of this dual-method approach.

## 5 Conclusions

This study provides further evidence to the still relatively small field of study focusing on ToP. We can again conclude many women feel their psychological well-being is secondary to their physical, reproductive health procedure which although many choose, many have to endure, often at the hands of cold and unsympathetic healthcare professionals. Furthermore, navigating a healthcare system to seek medical help for something which remains shrouded in social stigma and taboo, remains problematic for women, who often do not feel they are candidates for care or do not feel empowered to seek it, particularly if a woman is seeking a ToP by choice, in an increasingly prenatal society. This study adds further weight to calls demanding compassionate care for all women and birthing people, whilst respecting their reproductive health rights providing them the space and sentiment of unburdened reproductive justice, free from any form of caveat or condition. Finally, this study presents another example of innovative methodological interrogation, demonstrating how to handle a qualitative dataset with a *small-n* which is limited in data sources, but not in depth of meaning, whilst still rendering important results. The dual qualitative approach is something which should be considered by experimental qualitative practitioners to ensure a rigorous assessment of data is undertaken when data is sparse, but important and rich in detail. Taken together, we have not only demonstrated an advancement to the field of study, but offered an extension to the qualitative repertoire for those who intend to advance the methodological qualitative praxis with innovation.

## Data Availability

The datasets generated and/or analysed during this study are not publicly available due to the sensitive nature of the interviews, but a de-identified dataset may be available from the corresponding author upon reasonable request.

## Group members of The PUDDLES UK Collaboration

Amy Sampson^1^, Leonie Haddad^1^, Abigail Easter^1^, Peter von Dadelszen^1^, Davor Jurković^2^, Venetia Goodhart^2^, Rhiannon George-Carey^2^, Maria Memtsa^3^, Kayleigh S. Sheen^4^, Jane Sandall^1^, Laura A. Magee^1^, ^1^King’s College London, London, United Kingdom, ^2^University College London Hospitals NHS Foundation Trust, London, United Kingdom, ^3^Royal Free London NHS Foundation Trust, London, United Kingdom, ^4^University of the West of England, Bristol, United Kingdom
